# Alkaloids from *piper longum* protect dopaminergic neurons against inflammation-mediated damage induced by intranigral injection of lipopolysaccharide

**DOI:** 10.1186/s12906-016-1392-6

**Published:** 2016-10-24

**Authors:** Huan He, Wei-Wei Guo, Rong-Rong Xu, Xiao-Qing Chen, Nan Zhang, Xia Wu, Xiao-Min Wang

**Affiliations:** 1 Beijing Key Lab of TCM Collateral Disease Theory Research and School of Traditional Chinese Medicine, Capital Medical University, 10 Xitoutiao, Youanmen, Beijing, 100069 China; 2 Beijing Institute for Brain Disorders and Key laboratory of Neurodegenerative Diseases of the Ministry of Education, Capital Medical University, 10 Xitoutiao, Youanmen, Beijing, 10069 China

**Keywords:** Parkinson’s disease, Inflammation, Lipopolysaccharide, *Piper longum* L, Alkaloid

## Abstract

**Background:**

Alkaloids from *Piper longum* (PLA), extracted from *P. longum*, have potent anti-inflammatory effects. The aim of this study was to investigate whether PLA could protect dopaminergic neurons against inflammation-mediated damage by inhibiting microglial activation using a lipopolysaccharide (LPS)-induced dopaminergic neuronal damage rat model.

**Methods:**

The animal behaviors of rotational behavior, rotarod test and open-field test were investigated. The survival ratio of dopaminergic neurons and microglial activation were examined. The dopamine (DA) and its metabolite were detected by high performance liquid chromatography (HPLC). The effects of PLA on the expression of interleukin (IL)-6, interleukin (IL)-1β and tumor necrosis factor (TNF)-α were detected by enzyme-linked immunosorbent assay (ELISA). Reactive oxygen species (ROS) and nitric oxide (NO) were also estimated.

**Results:**

We showed that the survival ratio of tyrosine hydroxylase-immunoreactive (TH-ir) neurons in the substantia nigra pars compacta (SNpc) and DA content in the striatum were reduced after a single intranigral dose of LPS (10 μg) treatment. The survival rate of TH-ir neurons in the SNpc and DA levels in the striatum were significantly improved after treatment with PLA for 6 weeks. The over-activated microglial cells were suppressed by PLA treatment. We also observed that the levels of inflammatory cytokines, including TNF-α, IL-6 and IL-1β were decreased and the excessive production of ROS and NO were abolished after PLA treatment. Therefore, the behavioral dysfunctions induced by LPS were improved after PLA treatment.

**Conclusion:**

This study suggests that PLA plays a significant role in protecting dopaminergic neurons against inflammatory reaction induced damage.

## Background

Parkinson’s disease (PD) is one of the most common neurodegenerative disease, characterized by slow and progressive death of dopaminergic neurons in the substantia nigra pars compacta (SNpc) [1]. Although the mechanism of neuronal degeneration remains not throughly elucidated, increasing evidences suggest that neuroinflammatory processes may be involved in the progressive death of dopaminergic neurons [2–4]. Studies have shown that the activation of microglia plays a key role in neuroinflammation and a large quantity of reactive microglia was found in the SNpc of PD patient [5, 6]. Under normal circumstances, microglia typically exists in a resting state, which involved in immune surveillance and host defense against immunological stimuli. However, it comes to be activated when various pathogenic stimuli appear such as brain injury [7, 8]. Over-activated microglia produces various neurotoxic factors, such as nitric oxide (NO), tumor necrosis factor-alpha (TNF-α), interleukin-1β (IL-1β), prostaglandin E_2_ (PGE_2_), and reactive oxygen species (ROS), which lead to neuronal damage and results in a self-amplifying cycle of neuronal death [8, 9].

In the past, levodopa is the most widely used drugs for PD treatment, which mainly focused on dopamine (DA) compensation. But years of levodopa treatment causes motor complications, dyskinesia and its efficacy is counteracted [10]. Therefore, new drugs with novel mechanism for the treatment of PD are urgently needed. Several reports have shown that a number of traditional Chinese Medicine have neurotrophic and neuroprotective properties in PD animal models [11–13].

*Piper longum* L. (Piperaceae) has been used as traditional medicine in Asia and the Pacific Islands. Piperine is a main compound in *P. longum*. Alkaloids from *Piper longum* (PLA) are extracted from *P. longum* seed. It mainly contains 53.08 % piperine and 1.73 % piperlonguminine. Our previous work revealed that PLA possess neuroprotection function on dopaminergic neurons against 6-OHDA-induced damage and in the MPTP animal model of PD [14, 15]. Recently, another report showed that piperine and piperlonguminine protect rotenone-induced neuronal injury [16]. Besides, our previous work also showed that PLA significantly suppressed BV_2_ cells activation, attenuated expression of cyclooxygenase (COX)-2 and inhibited the excessive production of proinflammatory mediator IL-1β and PGE_2_ in LPS-induced BV_2_ cells [17].

Based on the previous studies, we hypothesized that PLA showed neuroprotective effects by reducing inflammation. In the present study, we used the classic lipopolysaccharide (LPS)-induced rat model of PD to examine whether PLA protects dopaminergic neurons against inflammation-mediated damage by inhibiting microglial activation.

## Methods

### PLA extract and reagents

*Piper longum* was purchased from Anguo, Hebei province, China, in 2014 and identified by Rong Luo, associate professor, School of Traditional Chinese Medicine, Capital Medical University. The voucher specimens of this material have been deposited in School of Traditional Chinese Medicine, Capital Medical University.

The PLA extract was made as our previous work described [15]. The content of total alkaloids was 74.6 % determined by UV, meanwhile the contents of piperine and piperlonguminine were 53.08 and 1.73 % respectively determined by HPLC. PLA was analyzed in a previous study carried out by our laboratory. Refer to this work for information about detailed compositions and chromatogram of PLA [15].

Lipopolysaccharide (LPS, from *Escherichia coli.* serotype O26:B6), dopamine (DA), 3,4-dihydroxyphenylacetic acid (DOPAC), mouse anti-tyrosine hydroxylase antibody, TritonX-100 and apomorphine were purchased from Sigma-Aldrich (St. Louis, MO, USA). Rabbit anti-Iba-1 antibody was purchased from BOSTER (Wuhan, China). Interleukin-1β (IL-6), interleukin-1β (IL-1β), tumor necrosis factor-alpha (TNF-α) and reactive oxygen species (ROS) enzyme-linked immunosorbent assay (ELISA) kits were purchased from MultiScience Biotech Co., Ltd (Hangzhou, China). Nitric oxide (NO) kit was purchased from NanjingJiancheng Bioengineering Institute (Jiangsu, China).

### Animals and surgery

Eighty adult male Sprague-Dawley rats (weight 260–300 g) were purchased from the Beijing Vital River Lab Animal Technology Co. Ltd. (Beijing, China) and maintained under standard conditions with a standard 12-h on/off light cycle, with food and water supplied *ad libitum*. After allowed to acclimate to their new surroundings for 1 week before experimental surgery, the rats were injected 2.0 μL LPS dissolved (5 mg/mL) in phosphate-buffed saline (PBS) into the right SNpc following a previous described protocol [18]. The injection position was anteroposterior −5.3 mm, lateral 2.0 mm and dorsoventral 7.8 mm from bregma. Sham-operated animals were injected 2 μL PBS into the right SNpc. Our reasearch had acquired the ethics approval by Animal Experiments and Experimental Animal Welfare Committee of Capital Medical University and all experimental procedures were approved by the Committee. The ethics approval number is AEEI-2014-081.

### Experiment design

The rats were randomly divided into five groups: the sham-operated group (*n* = 16), the LPS-injected group followed by vehicle treatment (model group, *n* = 16), the LPS-injected group followed by treatment with 25, 50 and 75 mg/kg PLA, respectively (*n* = 16 each group). Rats in three PLA treatment groups were intragastrically administered with PLA (dissolved in 0.5 % sodium carboxymethylcellulose) once a day after the surgery for 6 weeks. The sham-operated and model groups received 0.5 % sodium carboxymethylcellulose.

### Rotational behavior assay

On the second day after treatment with PLA for 3 and 6 weeks, the rats were injected hypodermically with 0.5 mg/kg apomorphine dissolved in physiological saline to examine the rotational behavior. The number of turns performed over 30-min testing period was counted.

### Rotarod test

All rats underwent a 3-day training program on a rotarod before the LPS injection, by which time a steady baseline level of rotarod performance was attained. Briefly, the rats were placed on the rod and sequentially tested at the speed accelerated from 0 to 40 rpm within 2 min. The time latency to fall from the rotarod at each speed level was recorded. At 3^th^ and 6^th^ week after treatment with PLA the rats were tested respectively.

### Open-field test

Rats in each group were tested by the Tru Scan activity monitoring system (Coulbourn Instruments), which contains acoustic insulation and lucifugal field (60 cm × 60 cm × 65 cm). Infrared device was installed at the top of the box, which was used to accurately track the movement and the behavior. After the end of each test, 75 % ethanol was used to thoroughly clean the open-field apparatus. The rat movement was recorded for the following parameters: total movement distance (cm), total movement time (s), total rest time (s) and horizontal velocity (cm/s). Each test time was 30 min.

### Preparation of tissue samples

Four rats from each group were randomly selected for morphological studies on the second day after the final behavioral tests. Decapitating all other rats, then the bilateral substantia nigra (SN) and striatum were rapidly dissected and stored at −80 °C. The SN was used for the quantification of proinflammatory cytokines, and the striatum for determination of the content of DA. For the morphological studies, rats were deeply anesthetized with chloral hydrate, then transcardially perfused with 200 mL saline followed by 200 mL of 4 % paraformaldehyde in 0.1 M phosphate buffer. Brains were removed and post-fixed in the same fixative and then immersed in a 20 % sucrose solution and a 30 % sucrose solution. Coronal section were cut on a freezing microtome (Leica, Germany) at a thickness of 40 μm and used for immunohistochemistry as described below.

### High performance liquid chromatography (HPLC)

The determination of DA and its metabolite 3,4-dihydroxyphenylacetic acid (DOPAC) was carried out using HPLC with a Coul Array electrochemical detector (Model 5600A, ESA, USA) equipped with Waters symmetry shield RP 18 column (150 × 3.9 mm, 5 μm). The mobile phase consisted of 50 mM sodium citrate, 8 % methanol, 0.1 mM EDTA · 2Na, 0.2 mM 1-octanesulfonic acid sodium salt and was finally adjusted to pH 4.1. The flow rate was 0.8 mL/min. Striatum tissues from 6 animals of each group were used and performed as described in previous work [19].

### Immunohistochemical staining of TH and Iba-1

Eight sections were selected for immunohistochemical staining of the tyrosine hydroxylase (TH). The mouse anti-TH antibody was diluted at 1:2000. Adjacent sections were used for detection of microglial marker Iba-1. The rabbit anti-Iba-1 antibody was diluted at 1:200. Sections were perforated with 0.3 % Triton-X 100 and blocked with normal horse serum (1:100 dilution), then were incubated with primary antibodies for 24 h at 4 °C. After that, sections were incubated with biotinylated anti-mouse antibody and biotinylated anti-rabbit antibody (Vector laboratories, Burlingame, CA, USA) respectively for 30 min at 37 °C, followed by avidin-biotin-peroxidase (Vector laboratories, Burlingame, CA, USA) incubation for 30 min at 37 °C. Finally, the immune complex was detected by 3, 3’ - diaminobenzidine (DAB).

To measure the numbers of TH-ir cells in the SN, stereological cell counting was performed. The optical fractionator method on a Stereo Investigator system (Micro Bright Field, USA) was used to count the total numbers of TH-ir neurons in the SN and a Leica microscope was used. The survival rate of TH-ir neurons in the SN was determined by counting the number of TH-ir neurons on LPS-injected side relative to the number of TH-ir neurons on the non-injected side. Quantitative analysis of Iba-1-stained immunohistochemical images were carried out with an Image-Pro Plus 6.0 system and positive results were expressed as average optical density value. All sections were coded and examined blindly.

### IL-6, IL-1β and TNF-α immunoassay

The right SN was used to detect the proinflammatory cytokines. Tissues were made into 10 % homogenate and then the homogenate was centrifuged at 3000 *g* for 15 min at 4 °C. The supernatant was collected at 4 °C. IL-6, IL-1β and TNF-α were detected using the commercial enzyme-linked immunosorbent assay (ELISA) kits (MultiScience Biotech, Hangzhou, China). All experimental procedures were performed according to the manufacturer’s instructions. Besides, we used the BCA protein kit to measure protein contents in samples according to the manual. The SN tissues from 6 animals of each group were used.

### Measurement of ROS and NO

The SN was also used to measure ROS and NO and tissues were made into 10 % homogenate as above. ROS was measured using enzyme-linked immunosorbent assay (ELISA) to measure absorbance value at 450 nm with ELISA kit. The content of NO was measured using NO assay kit according to the manufacturer’s guidelines by measuring the absorbance value at wavelength of 550 nm. BCA protein kit was used to measure the protein contents in samples according to the manual. The SN tissues from 6 animals of each group were used.

### Statistical analysis

Data were processed by commercially available software GraphPad Prism 5.0. Results are typically presented as means ± S.E.M. Statistical significance was assessed using a one-way analysis of variance (ANOVA), followed by Dunnett post hoc test (compares all the other columns with the designated control column). Significance was set at *P* < 0.05.

## Results

### PLA administration improves apomorphine-induced rotational behavior

**Fig. 1:**
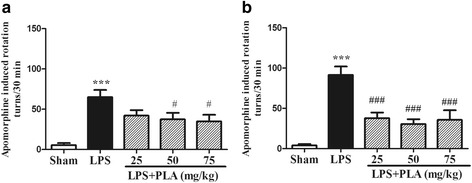
Effects of PLA treatment on apomorphine-induced rotational behavior. Rats were randomly grouped and then treated with PLA (25, 50 and 75 mg/kg) or vehicle for 6 weeks after LPS injection. On day 21 **a** and 42 **b**, rats were hypodermically injected with apomorphine (0.5 mg/kg) to induce rotational behavior. The number of turns were recorded for 30 min. *n* = 10–14. ^***^ *P* < 0.001 *vs.* sham group, ^#^ *P* < 0.05, ^###^ *P* < 0.001 *vs.* LPS group

The apomorphine-induced rotational cycles of all groups are shown in Fig. 1. The rotational cycles of model group animals increased than that of sham group (*P* < 0.001) examined both at 3^th^ and 6^th^ week after LPS injection. However, treatment with 25, 50 and 75 mg/kg PLA for 6 weeks significantly reduced the numbers of apomorphine-induced rotational turns compared with the model group (*P* < 0.001) (Fig. 1b).

### PLA administration improves rotarod behavior

**Fig. 2:**
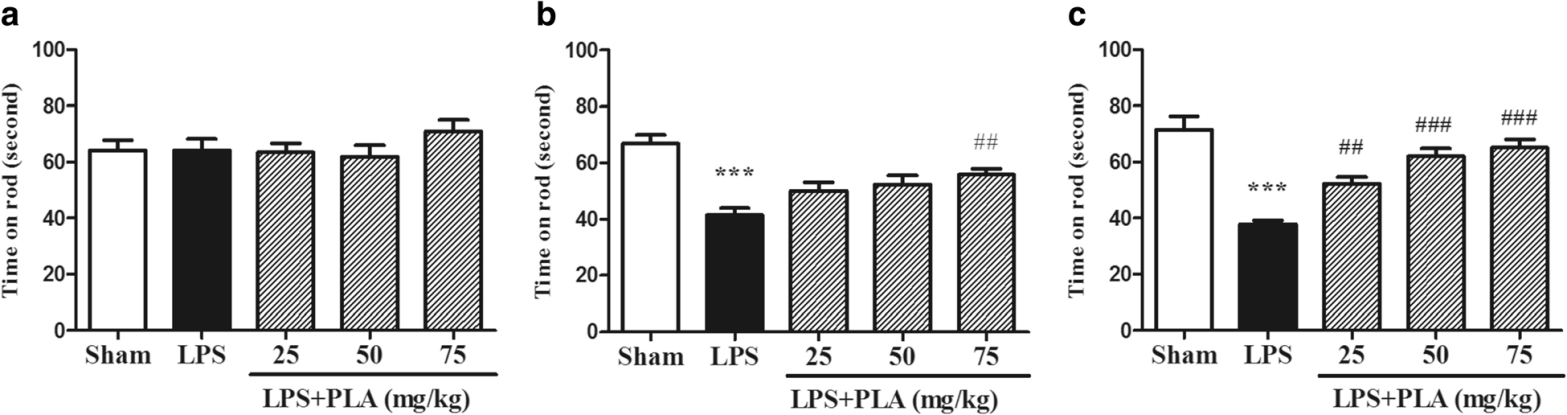
Effects of PLA treatment on rotarod behavior. Rats were randomly grouped and then treated with PLA (25, 50 and 75 mg/kg) or vehicle for 6 weeks after LPS injection. **a** A baseline trial was conducted before surgery. **b**, **c** The time each group animals remained on the rotarod after PLA administration for 3 and 6 weeks, respectively. *n* = 10–13. ^***^ *P* < 0.001 *vs.* sham group, ^##^ *P* < 0.01, ^###^ *P* < 0.001 *vs.* LPS group

As apomorphine-induced rotation test, the rotarod test is also a classic method to evaluate behavioral dysfunction of PD model rats. Animals in each group performed equally before LPS injection (Fig. 2a). A significant decrease of time which rats keep balance on rod was observed after LPS injection for 3 and 6 weeks (*P* < 0.001) (Fig. 2). However, treatment with PLA (25, 50 and 75 mg/kg) for 6 weeks may significantly improve the rotarod behavior (Fig. 2c).

### PLA administration improves locomotor activity

**Fig. 3:**
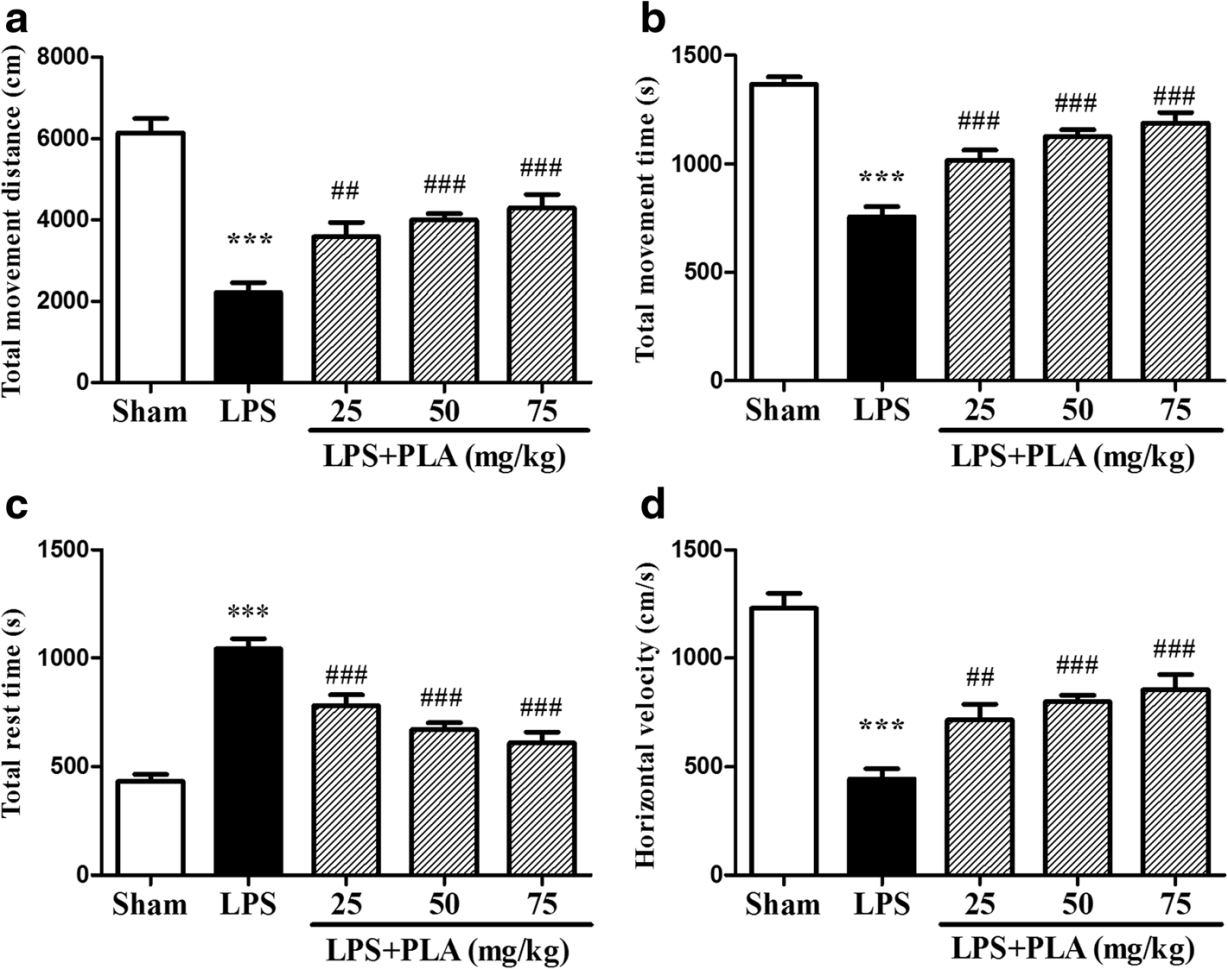
Effects of PLA treatment on locomotor activity. Rats were randomly grouped and then treated with PLA (25, 50 and 75 mg/kg) or vehicle for 6 weeks after LPS injection. On day 42, rats were placed into open-field apparatus, and spontaneous behaviors of 30 min was measured and analyzed by a Tru Scan 2.01 software. **a** total movement distance; **b** total movement time; **c** total rest time; **d** horizontal velocity. *n* = 10–11. ^***^ *P* < 0.001 *vs.* sham group, ^##^ *P* < 0.01, ^###^ *P* < 0.001 *vs.* LPS group

**Fig. 4:**
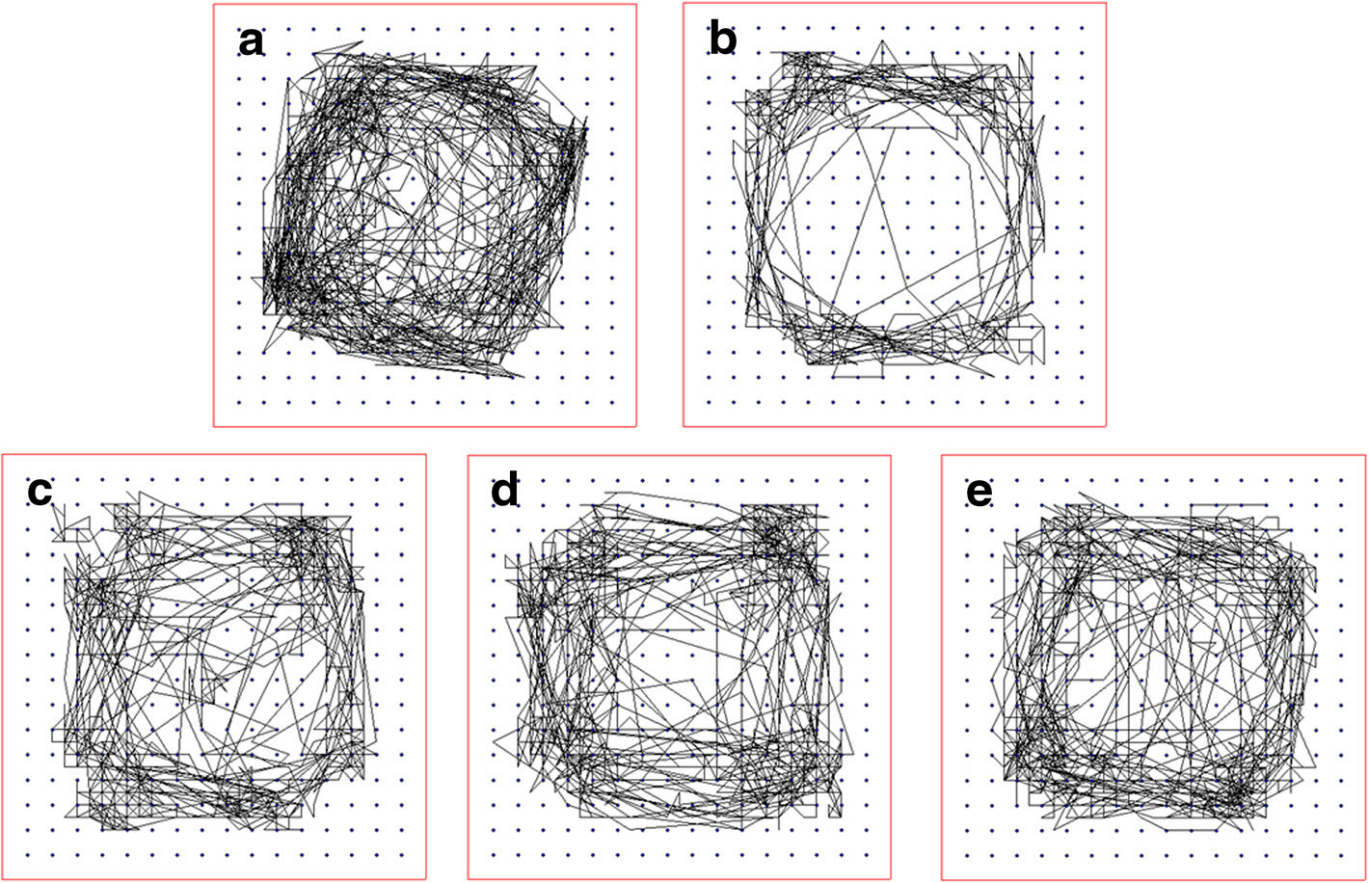
Effects of PLA treatment on the movement track of rats. **a** sham-operated group; **b** LPS-injected group followed by vehicle treatment; **c** the LPS-injected group followed by treatment with 25 mg/kg PLA; **d** the LPS-injected group followed by treatment with 50 mg/kg PLA; **e** the LPS-injected group followed by treatment with 75 mg/kg PLA

Open-field test is a classic behavioral test to comprehensively evaluate spontaneous behaviors of rats. The results showed that the most activities in the model group were significantly reduced than other groups. As shown in Fig. 3a, b, d, the activities of model group such as total movement distance, total movement time and horizontal velocity were all decreased. In contrast, the rest time for the rats in the model group was significantly higher than the others (Fig. 3c). Treatment with PLA (25, 50 and 75 mg/kg) for 6 weeks significantly increased the total movement distance, total movement time and horizontal velocity and decreased the rest time. Figure 4 shows the effect of PLA on the movement track of 30 min of rats in each group.

### PLA administration attenuates depletion of DA and DOPAC in the striatum induced by LPS intranigral injection

**Fig. 5:**
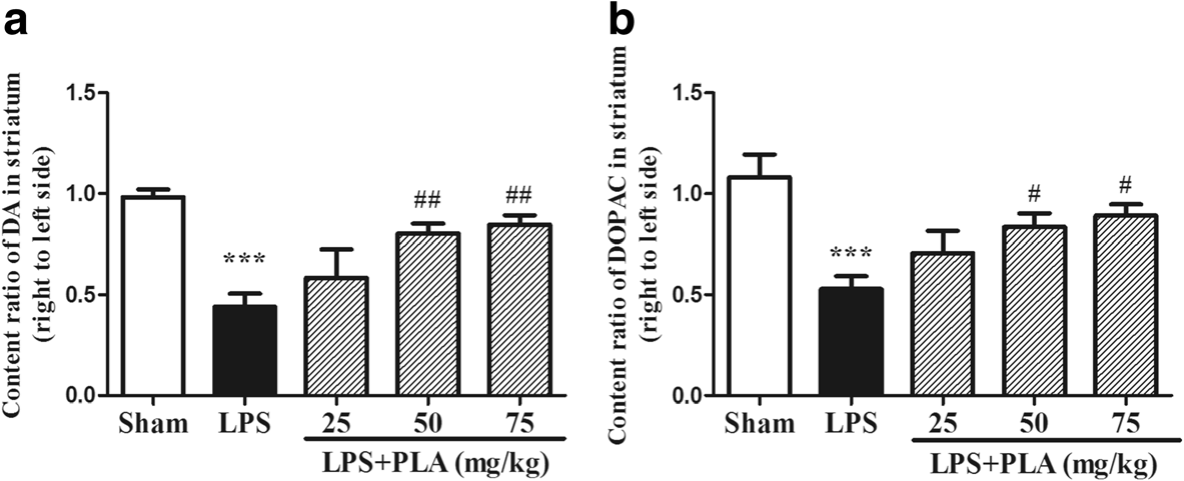
Effects of PLA treatment on the contents of DA and DOPAC in the striatum. Rats were randomly grouped and then treated with PLA (25, 50 and 75 mg/kg) or vehicle for 6 weeks after LPS injection. On day 42, rats were decapitated and the contents of DA and DOPAC in the striatum were detected by HPLC. The survival ratios (right relative to left side) of DA **a** and DOPAC **b** were calculated. *n* = 6. ^***^ *P* < 0.001 *vs.* sham group, ^#^ *P* < 0.05, ^##^ *P* < 0.01 *vs.* LPS group

The DA content in striatum was decreased after injection of LPS in the SN. In the model group, the survival levels of DA and DOPAC on the LPS-injected side were much lower than that of sham group (*P* < 0.001). After treatment with PLA 50 or 75 mg/kg for 6 weeks, the DA and DOPAC depletion in the striatum induced by LPS intranigral injection was significantly attenuated (Fig. 5).

### PLA treatment protects dopaminergic neurons from the injury induced by LPS intranigral injection

**Fig. 6:**
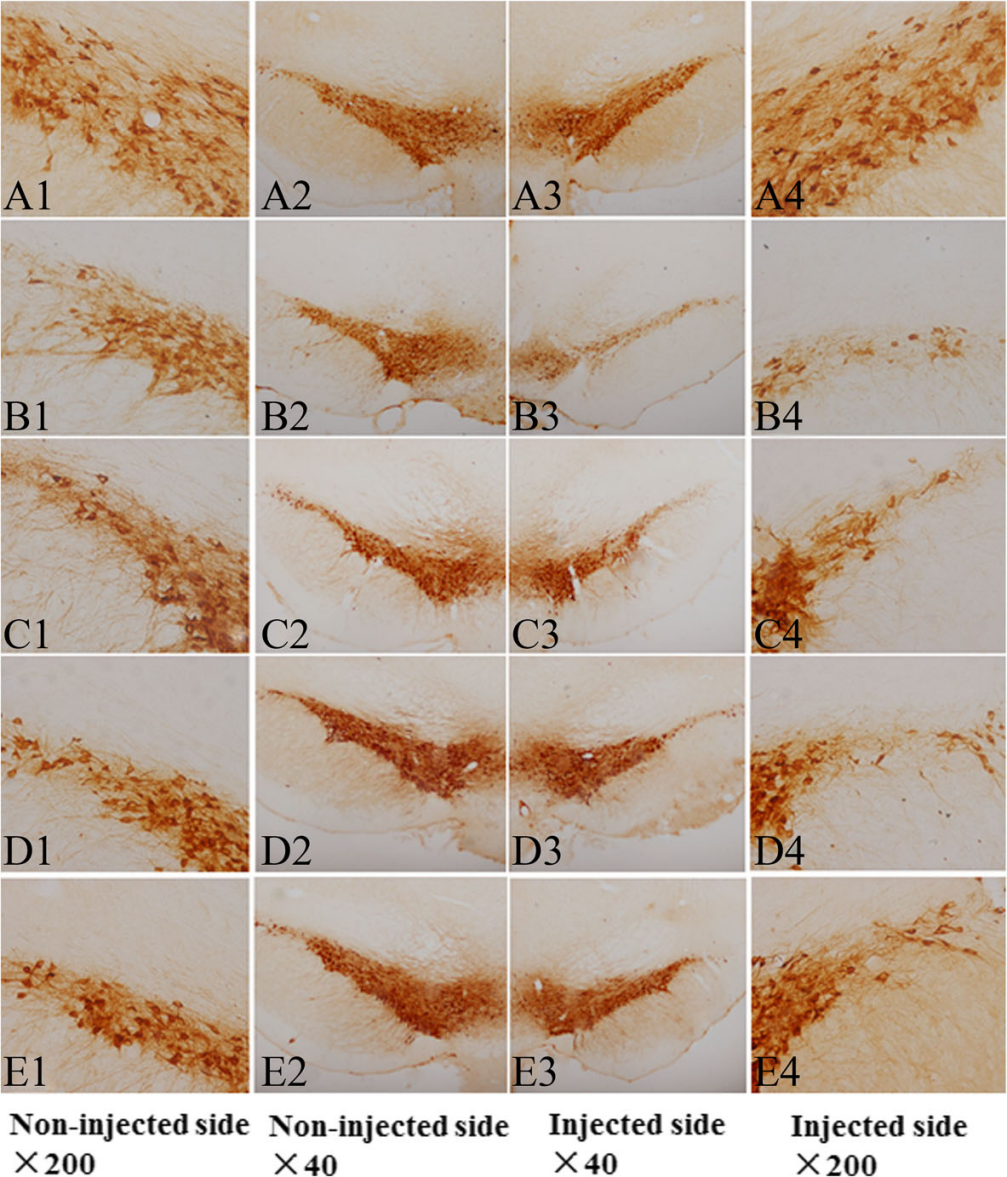
Morphological evidence of the protective effect of PLA against LPS-induced damage to dopaminergic neurons in the SN. Rats were randomly grouped and then treated with PLA (25, 50 and 75 mg/kg) or vehicle for 6 weeks after LPS injection. On day 42, rats were deeply anesthetized and transcardially perfused with 4 % paraformaldehyde and processed as above. Frozen sections at a thickness of 40 μm were cut and TH was detected by immunohistochemical staining to show dopaminergic neurons in the SNpc. (**A1**–**A4**) sham-operated group, TH staining was similar on the non-injected (A2) and injected (A3) side; (**B1**–**B4**) model group, LPS-injected followed by vehicle treatment, an obvious loss of TH-ir cells was seen on the injected side (B3); (**C1**–**C4**, **D1**–**D4**, and **E1**–**E4**) the LPS-injected group followed by treatment with 25, 50 and 75 mg/kg PLA respectively, much more TH-ir neurons survived on the LPS-injected side compared to model group

**Fig. 7:**
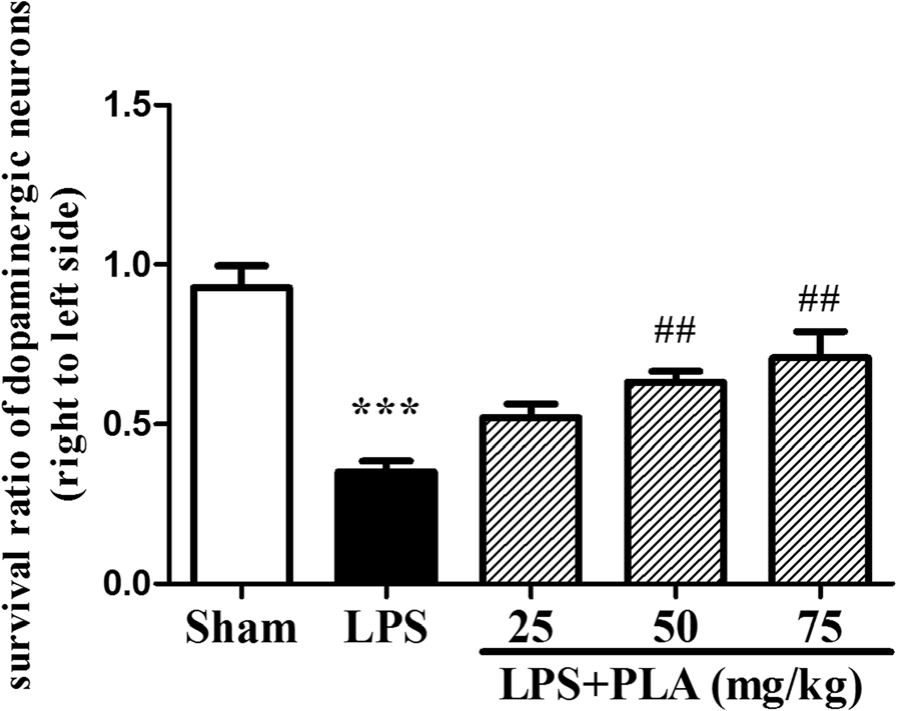
Effects of PLA treatment on the survival ratio of dopaminergic neurons in the SNpc. The numbers of TH-ir neurons in the SNpc were counted as described in methods. Survival ratio of the TH-ir neurons in the SNpc (the injected side relative to the non-injected side) was calculated. *n* = 4. ^***^ *P* < 0.001 *vs.* sham group, ^##^ *P* < 0.01 *vs.* LPS group

Representative microphotographs of TH immunohistochemical staining in the SNpc are shown in Fig. 6. The numbers of TH-ir neurons on the injection side and non-injection side were similar in sham-operated animals. Model group rats showed a marked loss of TH-ir neurons and their dendrites on the injection side. However, PLA (25, 50 and 75 mg/kg) treatment significantly recovered this loss of nigral TH immunoreactivity. The survival ratio of dopaminergic neuron on the LPS-injected side relative to the non-injected side was shown in Fig. 7. Compared with the sham group, the survival of TH-ir neurons in the SNpc of model group was significantly decreased (*P* < 0.001). In contrast, the rats treated with 50 and 75 mg/kg PLA after LPS injection showed a great increase in the survival rate of TH-ir neurons in the SNpc when compared to model group rats (*P* < 0.01). A low dose (25 mg/kg) of PLA showed an obvious trend toward increasing the survival of TH-ir neuros in the SNpc, although not statistically significant.

### PLA treatment inhibits microglial activation in the SNpc induced by LPS intranigral injection

**Fig. 8:**
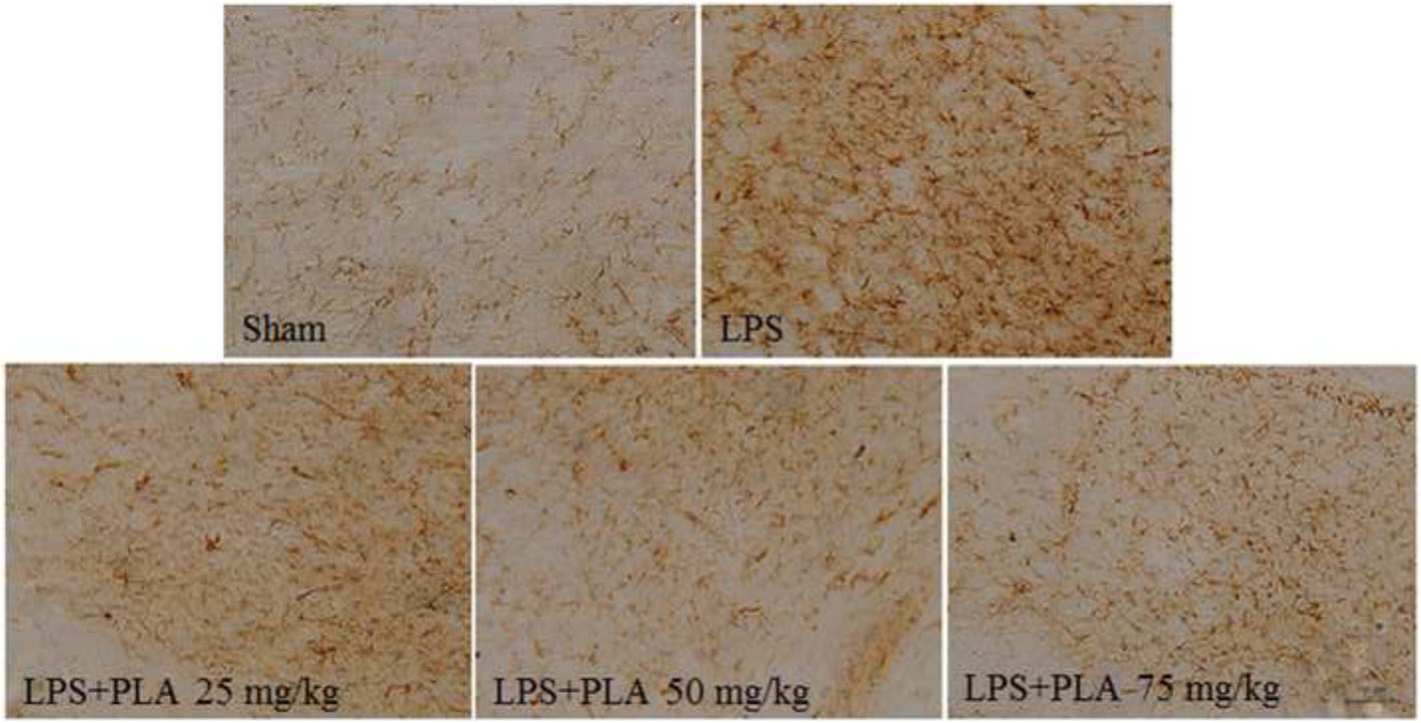
PLA treatment inhibits microglial activation. The morphological changes of microglia in the SNpc were revealed by Iba-1 immunostaining. Representative micrographs were shown. Scale bar represents 100 μm

**Fig. 9:**
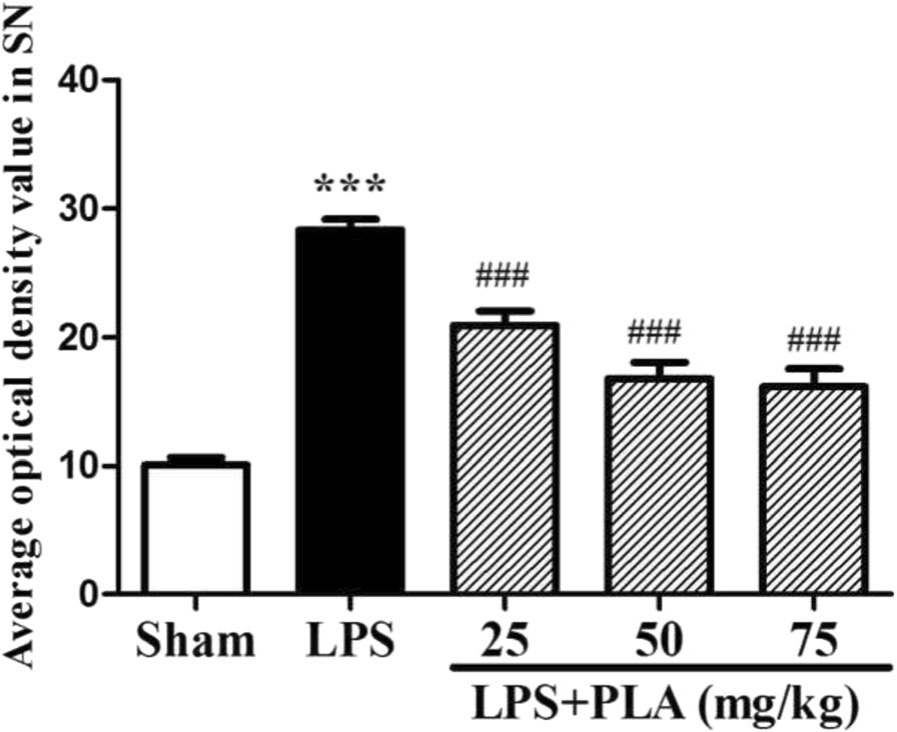
Effects of PLA treatment on the average optical density of Iba-1 in the SNpc. *n* = 4. ^***^ *P* < 0.001 *vs.* sham group, ^###^ *P* < 0.001 *vs.* LPS group

Immunocytochemical staining of ionized calcium binding adaptor molecule-1 (Iba-1) antibody was used to reveal microglial activation. As shown in Fig. 8, microglia underwent a morphological change from resting state to activated cells with larger cell body and branching coarsening in the SNpc after LPS injection. However, the activation of microglia was significantly suppressed after PLA treatment. The average optical density value was shown in Fig. 9. Compared with the sham group, the Iba-1 content increased significantly in the LPS injection group (*P* < 0.001), but which was significantly decreased after treatment with PLA for 6 weeks (*P* < 0.001).

### PLA treatment inhibits the release of TNF-α, IL-6 and IL-1β in the SN induced by LPS intranigral injection

**Fig. 10:**
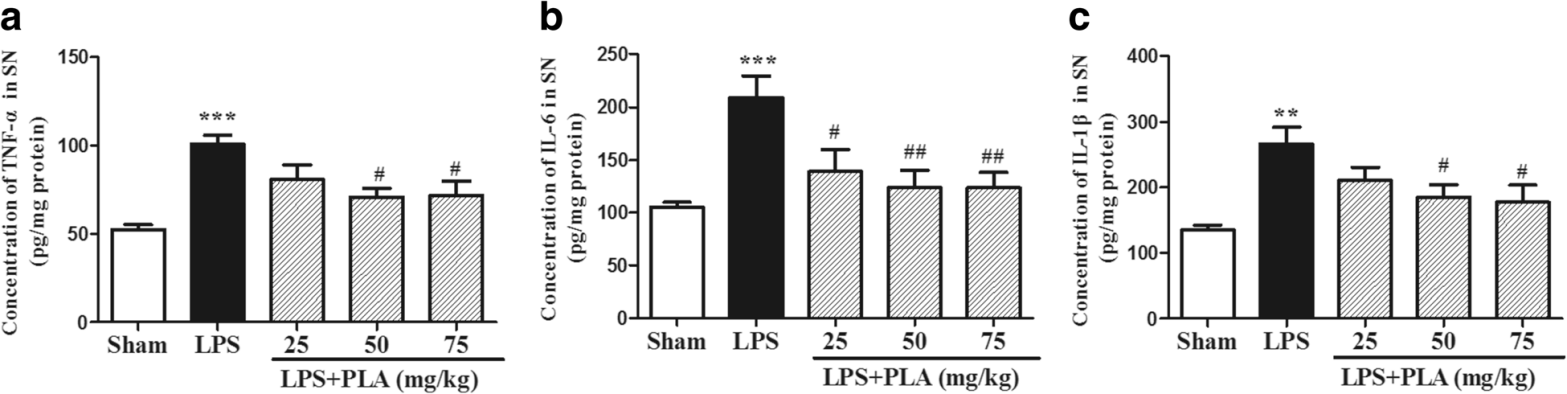
Effects of PLA treatment on the concentrations of proinflammatory cytokines in the SN. Rats were randomly grouped and then treated with PLA (25, 50 and 75 mg/kg) or vehicle for 6 weeks after LPS injection. On day 42, rats were decapitated and the SN was used to detect the concentrations of TNF-α **a** IL-6 **b** and IL-1β **c** by commercial ELISA kit. *n* = 6. ^**^ *P* < 0.01, ^***^ *P* < 0.001 *vs.* sham group, ^#^ *P* < 0.05, ^##^ *P* < 0.01 *vs.* LPS group

Intranigral injection of LPS induced excessive release of proinflammatory cytokines. The concentrations of proinflammatory cytokines TNF-α, IL-6 and IL-1β in the SN of model group rats were significantly increased when compared with the sham-operated group rats (*P* < 0.01). Treatment with PLA 50 and 75 mg/kg for 6 weeks significantly decreased the concentrations of TNF-α, IL-6 and IL-1β when compared with model group (*P* < 0.05). Rats treated with 25 mg/kg PLA, only the IL-6 concentration was decreased significantly compared with model group (*P* < 0.05) (Fig. 10).

### PLA treatment inhibits the release of ROS and NO in the SN induced by LPS intranigral injection

**Fig. 11:**
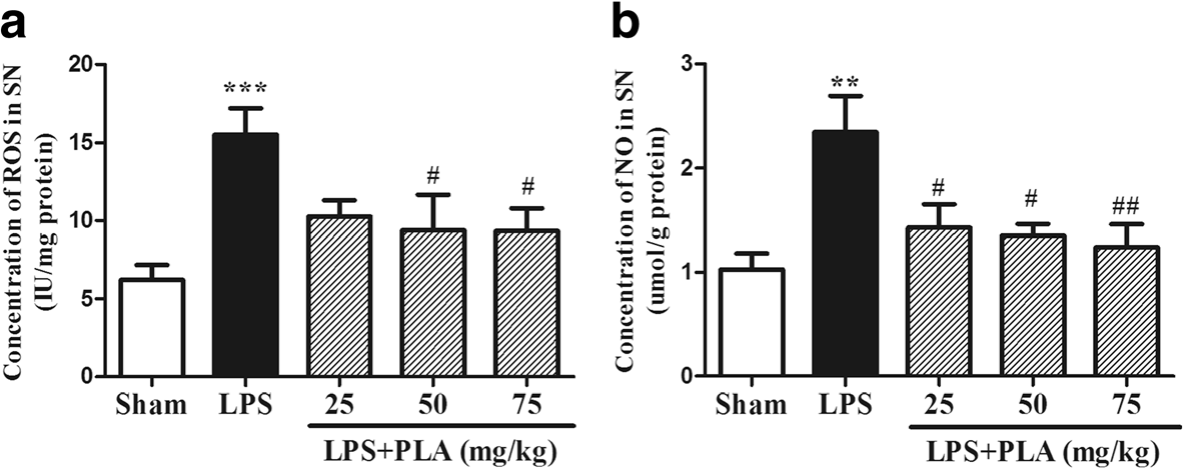
Effects of PLA treatment on the concentrations of ROS and NO in the SN. Rats were randomly grouped and then treated with PLA (25, 50 and 75 mg/kg) or vehicle for 6 weeks after LPS injection. On day 42, rats were decapitated and the SN was used to detect the concentration of ROS **a** by commercial ELISA kit and the concentration of NO **b** by NO assay kit. *n* = 6. ^**^ *P* < 0.01, ^***^ *P* < 0.001 *vs.* sham group, ^#^ *P* < 0.05, ^##^ *P* < 0.01 *vs.* LPS group

To demonstrate if PLA protecting the dopaminergic neurons is related to inhibiting the release of ROS and NO, the concentrations of ROS and NO in the SN were observed. The data showed that the concentrations of ROS and NO in model group rats were markedly increased compared with sham-operated group rats (*P* < 0.01). The concentrations of ROS and NO of rats treated with 50 and 75 mg/kg PLA for 6 weeks were fewer than that of rats in model group (*P* < 0.05). Rats treated with 25 mg/kg PLA, only the NO concentration showed a significant decrease compared with model group (*P* < 0.05) (Fig. 11).

## Discussion

A number of researches have suggested that inflammatory process plays a significant role in the progression of PD, especially microglial activation [8, 9, 20]. LPS, a component of the cell wall of Gram-negative bacterial, is extensively used to induce inflammation by activate glial cell [18, 19]. The PD models induced by LPS both in vitro and in vivo are widely used, which can not only reflect the role of neuroinflammation in PD but also have been used in drug discovery [21–23]. For example, pioglitazone and naloxone have been studied for their potential neuroprotective effects on LPS-induced PD models [24, 25]. It was reported that, the inflammatory reaction induced by injecting LPS into SN had a selective irreversible and long lasting damage on the dopaminergic neurons, which ultimately resulted in neurodegeneration [20, 22, 26]. Many studies also showed that a single intranigral injection of LPS led to depletion of DA and loss of dopaminergic neurons in animals [21, 27, 28].

In the present study, the effect of PLA on LPS induced rat model was examined and three different behavioral tests were conducted. The apomorphine-induced rotational test was used to estimate the injury degree of dopaminergic system [29]. Apomorphine-induced rotation cycles signifcantly increased in the model group rats induced by intranigral injection of LPS, in contrast, PLA treatment showed an improvement effect on this behavioral dysfunction (Fig. 1). The other two behavioral dysfunctions were also significantly improved by PLA treatment measured by rotarod test and open-field test. Further studies suggested that PLA treatment was able to block the loss of TH-ir neurons in the SNpc in LPS intranigral injection rats.

Studies have shown that microglial cells are readily activated and a variety of neurotoxic factors exist in the SN of LPS-induced PD model rats, which cause the damage of dopaminergic neurons [30–33]. The neurotoxic factors include IL-6, IL-1β, TNF-α, O_2_^−^, NO and so on [33, 34]. Our previous work suggested that PLA could significantly inhibit LPS-induced BV_2_cell activation and proinflammatory mediator production, such as IL-1β [17]. In the present study, our results showed that an intranigral injection of LPS led the microglial over-activation and the production of a large amount of neurotoxic proinflammatory factors (TNF-α, IL-6 and IL-1β), which triggered the cascade of events and caused the death of neighboring dopaminergic neurons, in contrast, PLA treatment was able to suppress the activation of microglia and block the release of these proinflammatory cytokines, attenuate neuroinflammation, and protect dopaminergic neurons damage. Given our previous work that PLA could cross the blood-brain barrier [35], these data indicates that PLA has potent anti-inflammatory activity in the central nervous system.

Nuclear factor κB (NF-κB) signaling pathway is the most significant pathway which mediates the LPS-induced microglial inflammatory response and regulates the production of various inflammatory mediators [29, 30]. In our previous work, the role of PLA in modulating NF-κB pathway in BV_2_ cells was studied [17]. PLA could inhibit the nuclear translocation of p65 subunit of NF-κB. The p65 protein level in the nucleus was increased after LPS stimulation, however PLA treatment significantly counteracted it. What’s more, PLA could also inhibit NF-κB activity through the inhibitory effect on the degradation of IκB. Thus, the inhibitory effect on NF-κB activitation might be involved in the anti-inflammatory properties of PLA.

In microglial activation, ROS production occurs prior to the cytokine production and NO is another neurotoxic factor [33, 36]. A recent study showed that alkaloids from *P. longum* decreased ROS production, stabilized mitochondrial membrane potential and inhibited the opening of the mitochondrial permeability transition pore (mPTP) [16]. In this study, we further investigated the effects of PLA on production of neurotoxic factors ROS and NO induced by LPS intranigral injection. Our results indicated that PLA could reduce the production of ROS and NO induced by LPS, which might be a significant mediator of the neuroprotective effect of PLA. Taking our previous work together, PLA might be a multi-component and multi-target approach in the treatment of PD. Further studies are needed to better understand the mechanism involved in PD treatment.

## Conclusion

In this study, the treatment of PLA, an active extract of the traditional Chinese medicine *P. longum*, was able to attenuate the depletion of DA and DOPAC in the striatum, facilitate the survival of damaged neurons by inhibiting microglial activation and suppressing the release of neurotoxic factors such as TNF-α, IL-1β, IL-6, ROS and NO, and improve the LPS-induced behavioral dysfunctions. In summary, this study suggests that PLA may have a protective effect on dopaminergic neurons against inflammatory reaction induced damage.

## Abbreviations

6-OHDA: 6-hydroxydopamine
DA: Dopamine
DOPAC: 3, 4-dihydroxyphenylacetic acid
HPLC: High performance liquid chromatography
Iba-1: Ionized calcium binding adaptor molecule-1
IL-1β: Interleukin-1β
IL-6: Interleukin-6
IκB: Inhibitor of NF-κB
LPS: Lipopolysaccharide
MPTP: 1-methyl-4-phenyl-1,2,3,6-tetrahydropyridine
NF-κB: Nuclear factor κB
NO: Nitric oxide
PD: Parkinson’s disease
PGE_2_: Prostaglandin E_2_
PLA: Alkaloids from *Piper longum*
ROS: Reactive oxygen species
SN: Substantia nigra
SNpc: Substantia nigra pars compacta
TH: Tyrosine hydroxylase
TH-ir: Tyrosine hydroxylase-immunoreactive
TNF-α: Tumour necrosis factor-α
